# Biomarkers of Oxidative Stress in Acute and Chronic Diseases

**DOI:** 10.3390/antiox11091766

**Published:** 2022-09-07

**Authors:** Luca Massaccesi, Carmela Rita Balistreri

**Affiliations:** 1 Department of Biomedical Sciences for Health, Università degli Studi di Milano, Via Mangiagalli, 31, 20133 Milan, Italy; 2 Cellular and Molecular Biology and Clinical Pathology Laboratory, Department of Biomedicine, Neuroscience and Advanced Diagnostics (Bi.N.D.), University of Palermo, Corso Tukory 211, 90134 Palermo, Italy

Molecular biomarkers consent to apply individual decisions in the complex management of both acute or chronic diseases, and their identification constitutes a fundamental phase for achieving the important object to develop personalized therapies. Pathways and molecules involved in the pathophysiological mechanisms of acute or chronic diseases, i.e., oxidative stress (OS), might represent optimal candidates for biomarkers. Oxidative stress constitutes a crucial mechanism in the onset and progression of various chronic inflammatory diseases, including cardiovascular diseases, neurodegenerative diseases, infection diseases, diabetes, and cancer. The loss of physiological balance between pro-oxidant factors and antioxidant defenses, fundamental for both the maintenance of low levels of free radicals and the homeostasis of human tissues, leads to excessive production of reactive oxygen species (ROS) and reactive nitrogen species (RNS), which are toxic for cells. This determines the cell damage that accumulates, leading to the destruction of cellular homeostasis with a severe relapse reaching the systemic level. The growing understanding of redox dynamics and the biological mechanisms underlying diseases, and the need to have increasingly early biomarkers in for optimizing the diagnostic process, have led to the development of new specific and sensitive instruments for the OS measurement in different biological materials. Many of these new biomarkers have been proposed as effective tools in the monitoring of different diseases, and as valuable aids in the evaluation of the effectiveness of treatments. Accordingly, OS biomarkers are relevant in the evaluation of the disease status and the health-enhancing effects of antioxidants.

Our Special Issue presents innovative research [1,2,3,4,5,6,7,8,9,10,11,12] and review [13,14,15] papers addressing many of these questions related to the fundamental OS involvement in the onset and progression of several acute and chronic diseases, and about the correlated biomarkers and potential therapies. Precisely, the 12 research articles [1,2,3,4,5,6,7,8,9,10,11,12] included in this Special Issue are focused on evidencing the OS role in diagnostics, signatures of the disease course, molecules involved, OS potential treatments and effects, and short- and long-term consequences. Precisely, Chen et al. [1], by performing a nano-electrospray ionization–mass spectrometry (nanoESI-MS)-based lipidomic profiling study on lipid accumulation and oxidation in kidney cells in the chronic kidney disease (CKD) model, confirmed the key role of OS and lipid dysregulation in renal lipid droplet (LD) alteration at the molecular level. Consequently, Chen and coworkers underlined that the lipid hydroperoxides, especially TGOOH and PCOOH species, can have the role as diagnostic CKD biomarkers. Thus, their data have provided new insight into CKD development from the view of the LD lipid metabolism.

Of note, also, is the article from Peruzzi and coworkers [2], where they elegantly documented how the monitoring of urinary malondialdehyde (MDA) levels can be used a useful tool for the management of patients affected by congenital central hypoventilation syndrome (CCHS), a rare genetic disorder of the autonomic nervous system, and especially of the respiratory control during sleep, characterized by unavailable drug therapy, and case survival dependent only on permanent ventilatory support during sleep. Thus, Peruzzi and coworkers used these data as a smart disclosure to the CCHS scientific community that CCHS is associated with a higher systemic oxidative status, and urinary MDA represents the key biomarker of oxidation. Precisely, the monitoring of the level of impairment of systemic oxidative status could help stratify the risk in CCHS patients.

Shukry and colleagues [3] evidenced, by testing 35 Wistar albino rats, the beneficial effects of Ornipural^®^, a therapeutic solution containing several active ingredients, against the toxic action of malathion, oxidative agents, and inflammatory mediators, by restoring biochemical parameters, enhancing antioxidant defense mechanisms, and reducing the generation of inflammatory mediators. Kukolj et al. [4], by using Y59 rats injected intraperitoneally with iron dextran solution at a dose of 50 mg/kg or exposed to inhaled anesthetics sevoflurane and isoflurane and their combination for 28 days every other day, showed that the anesthetics reduce the rat’s organ weight and increases OS in peripheral tissues, leading to M1 macrophage polarization. Consequently, these discoveries suggested that the iron, in combination with sevoflurane, has a protective effect in tissues showing the M2 phenotype of macrophages, while the combination of iron dextran and isoflurane in rats determines an increase in the erythropoiesis process via induction of hypoxia. Similarly, Nilolava et al. [5] demonstrated, for the first time, the protective therapeutic effects of extracts of *Lemna minor* (*L. minor*, *Duckweed*; a fast-growing freshwater plant used in traditional medicine as an antiscorbutic, depurative diuretic, natural agent effective for colds) on bleomycin-induced pulmonary fibrosis in mouse models. Moreover, they examined the mechanism used by *L. minor* extract action against pulmonary fibrosis. Thus, they hypothesized that *L. minor* prevents bleomycin-induced lung disorders and fibrosis in mice model by regulating levels of protein carbonylation and protein peroxidation, inhibiting the production of proinflammatory, pro-fibrous cytokines, and reducing oxidative disorders.

Liou and coworkers [6] by investigating on the causes determining birth asphyxia, a physiological derangement seen in newborn infants due to a prolonged or profound mismatch between oxygen demand and oxygen delivery, and on its complications, such as the severe neurodevelopmental disabilities, as the syndrome of hypoxic-ischemic encephalopathy (HIE) having multi-organ involvement, demonstrated that the first blood glucose level is a significant biomarker for clinical staging, MRI findings, hearing impairment, and neurodevelopmental outcomes in neonatal HIE.

Moreover, in a recent longitudinal study in rural Senegal, Zhang, and coworkers [7], examined the relationship between blood pressure measures and c reactive protein (CRP) and MDA levels determined in dried blood spots (DBS), including whether baseline levels of CRP or MDA were associated with changes in BP observed over a 1-year period. They showed that among women with incident hypertension living in rural Senegal, DBS CRP, a biomarker of chronic inflammation, was significantly associated with systolic BP (SBP) change over a 1-year period; while DBS MDA, a biomarker of acute oxidative stress, was significantly associated with concurrent SBP levels.

Interestingly, Cicero et al. [8] performing a pilot study, and using ^1^H-NMR spectroscopy, evidenced a typical metabolic profile characterized by a significant increment in circulating KB, ALCAR, glutamate, and Phe/Tyr ratio, and a decrease in total amino acids, histidine, arginine, alanine, and methionine, in patients affected by Takotsubo syndrome. Based on this result, they can envisage that the future inclusion of metabolic markers in clinical practice might improve risk stratification in TTS.

Of note are, also, the results of two research articles [10,12], which demonstrated the key role of OS in non-alcoholic steatohepatitis (NASH), Duchenne muscular dystrophy (DMD) and their complications. Precisely, Hui et al. [10], in the NASH model mice and by conducting a comprehensive lipidomic analysis covering both intact lipids and lipid hydroperoxides in the liver and kidney tissues using liquid chromatography–mass spectrometry (LC/MS), demonstrated that OS played a crucial role in lipid dysregulation and peroxidation at the molecular level. Thus, their data provided a better understanding of OS in NASH development from the view of lipid metabolism, providing direct evidence for the oxidative damage-caused lipid hydro-peroxidation in NASH. Terrill et al. [12] elegantly documented by using the mdx mouse model of DMD that plasma Cys34 albumin thiol oxidation is a useful blood biomarker of OS in mdx mice, and changes in such thiol oxidation in plasma more closely reflect changes in protein thiol oxidation in dystrophic muscle, than the commonly used biomarker protein carbonylation. Thus, the data presented in this work suggest that albumin Cys34 thiol oxidation has the potential to be a useful robust blood biomarker of dystro-pathology and may be used to efficiently evaluate and advance the development of therapies for DMD.

Another research article reports the interesting results obtained from Guseva Canu et al. [9] in developing a new method, the LC–ESI-MS/MS method, for MDA detection and quantification in exhaled breath condensate (EBC) by using DNPH as the derivatizing agent. The developed method presented acceptable performances, and was successfully applied to 164 EBC samples, demonstrating its accuracy and precision. Thus, the authors proposed to use this method in future epidemiological studies focusing on comparing different volunteers or groups of subjects for oxidative stress in the respiratory system.

In another research article, Sienko and colleagues [11] stressed how the assessment of OS biomarkers can be advantaged for evaluating the status of kidneys for transplant preserved in static cold storage (SCS) (group 1) or hypothermic machine perfusion (HMP) (group 2), and their function when transplanted.

This Special Issue also gathered three review papers [13,14,15] that are focused on evidencing the key role of OS as fundamental mechanism of the pathogenesis of osteomyelitis, aorta diseases, and Alzheimer disease. Massaccesi and coworkers described [13] the very important role of OS in the osteomyelitis disease and its consequences on the delicate balance between osteoblastogenesis and osteoclastogenesis. They have, therefore, emphasized the possibility of measuring the level of OS more accurately as a valid support in the management of the disease. The review from Balistreri and coworkers [14] stresses the key role of OS in the pathophysiology of aorta diseases and describes emerging OS pathways as potential biomarkers of such diseases, as well as potential solutions, approaches, and treatments. Moreover, Buccellato et al. [15] summarized the key role of OS in Alzheimer disease (AD) and underlined how oxidative damage products may be used as possible peripheral biomarkers in AD, and in the preclinical phases of the disease.

We would like to thank all the authors who contributed to this Special Issue, (1) for having provided new insights into the OS role in diverse diseases and (2) about OS products or related pathways to use as innovative biomarkers, and their knowledge on (3) OS potential treatments and effects, and short- and long-term consequences. Despite the relevance of the topics covered in the papers published in this Special Issue, many aspects remain relatively limited. However, we hope to have provided the corrected basis for initiating novel and sophisticated investigations on the topics described by all the authors, and thanks them for the support of the innovative omics technologies, for obtaining data at multi-levels (i.e., genomics, epigenomics, proteomics, metabolomics, etc.) useful for application in the clinical management of acute or chronic diseases.
